# Gastrointestinal stromal tumors with atypical features and presentations: A case series

**DOI:** 10.1016/j.ijscr.2025.111569

**Published:** 2025-06-26

**Authors:** Alazar Berhe Aregawi, Bizunesh Dires Fenta, Mekashaw Altaseb Worku, Teketel Tadesse Geremew

**Affiliations:** a Department of Surgery, Hawassa University Comprehensive Specialized Hospital, Hawassa, Sidama, Ethiopia; b Department of Pathology, Hawassa University Comprehensive Specialized Hospital, Hawassa, Sidama, Ethiopia; c Department of Surgery, Bensa Daye General hospital, Sidama, Ethiopia

**Keywords:** Mesenchymal tumors, Small bowel tumors, Intussusceptions, Intrabdominal mass, Mesenteric tumor, Acute abdomen, Case series

## Abstract

**Introduction and importance:**

Gastrointestinal stromal tumors (GISTs) are the most common mesenchymal tumors to affect the digestive tract. These tumors arise from the interstitial cells of Cajal. Although the majority of GISTs occur in the stomach, they can arise anywhere along the GI tract, including the small intestine, colon, esophagus, and very rarely from extra intestinal locations. GISTs mainly affect older adults, with a slight male preponderance. Small size GISTs can be discovered incidentally during imaging or endoscopy. Symptoms vary depending on the location and size of the tumor, those related to the tumor mass effect (abdominal pain, discomfort, distension and a palpable mass), or others presenting with anemia and GI hemorrhage. Contrast-enhanced abdomino-pelvic CT scan is the investigation of choice for staging and follow-up. GISTs are distinguished from other tumors using immunohistochemical test positivity for CD 117and CD 34.

Surgery is the standard treatment for localized GISTs followed by risk stratification. Adjuvant imatinib should be considered in all patients with significant risk of recurrence following resection of primary GISTs.

**Case presentation:**

Here we describe a series of GISTs presenting at atypical locations, unique presentations, and atypical histologic variants. The first case was a 35-years old female patient who underwent an emergency laparotomy for small bowel obstruction and later diagnosed to have a mesenteric GIST (an extraintestinal GIST). The second case was a 60-year-old male patient who underwent an emergency laparotomy for a small bowel obstruction secondary to an intrabdominal mass and later diagnosed to have a jejunal GIST. The third case was a 72 years old male patient who underwent exploratory laparotomy for gastro-gastric intussusception secondary to gastric GIST. The fourth case was a 55 years old male patient who underwent exploratory laparotomy for gastric GIST with liver secondaries. In immunohistochemistry examination, all were positive for CD 117.

**Clinical discussion:**

Clinical presentation of GISTs depend on the tumor size and location. Small tumors are usually asymptomatic. If symptomatic, the most common clinical manifestations are features of anemia, GI bleeding, compressive symptoms, and intestinal obstruction. The diagnosis of GIST is confirmed using tissue histology and immunohistochemistry tests.

**Conclusion:**

Even though GIST constitutes the smallest portion of GI neoplasms, physicians need to have high index of suspicion for it in patients presenting with vague abdominal pain and consider abdominopelvic CT as part of patients' work up.

## Highlights

• Gastrointestinal stromal tumors (GISTs) are the most common mesenchymal tumors to affect the digestive tract.
• Here we describe a series of GISTs presenting at atypical locations, unique presentations, and atypical histologic variants.
• The first case was a 35-years old female patient who underwent an emergency laparotomy for small bowel obstruction and later diagnosed to have a mesenteric GIST (an extraintestinal GIST).
• The second case was a 60-year-old male patient who underwent an emergency laparotomy for a small bowel obstruction secondary to an intrabdominal mass and later diagnosed to have a jejunal GIST.
• The third case was a 72 years old male patient who underwent exploratory laparotomy for gastro-gastric intussusception secondary to gastric GIST.
• The fourth case was a 55 years old male patient who underwent laparotomy for gastric epithelioid GIST with liver secondaries. In IHC examination, all were positive for CD 117.

## 1 Introduction

Gastrointestinal stromal tumors (GISTs) are rare mesenchymal tumors representing less than 1 % of all gastrointestinal (GI) neoplasms [[1], [2], [3]]. GISTs commonly arise from the stomach (60 %) and small intestine (20 %–30 %), other locations are the large intestine (5 %), and the esophagus in 2 %–5 % of cases [1,4,5].

Clinical presentation of GISTs depend on the tumor size and location [2]. small tumors are usually asymptomatic. If symptomatic, the most common clinical manifestations are features of anemia, GI bleeding, compressive symptoms, and intestinal obstruction [2,5,6]. Because of the non-specific symptoms making a preoperative diagnosis of GIST is often challenging until complications arise resulting in high morbidity and mortality [2,7].Radiologic studies are highly helpful but are not 100 % certain [2]. The diagnosis of GIST is confirmed using tissue histology and immunohistochemistry tests [8].

GISTs are acquired by gain-of-function mutations in the proto-oncogene receptor tyrosine kinases that regulates the interstitial cells of cajal (ICC). Activating mutations in KIT being the most frequent form. The other is mutation in PDGFRA. These mutations are noted in 85–90 % of GISTs. The absence of these mutations makes the diagnosis of GIST challenging [3,6,[8], [9], [10]]. GISTs have three histologic variants, spindle type (70 %), epitheloid (20 %) and mixed variant (10 %) [3].

Surgical remains to be the gold standard treatment of GIST and post-surgical histopathologic assessment is essential to guide further management and for prognostication [2,7]. Adjuvant tyrosine kinase inhibitors like imatinib mesylate therapy significantly improves patient's survival if the tumor is metastatic, unresectable, or recurrent [2,10,11]. The aim of this case-series is to make physicians aware of this relatively rare pathology pertaining to patient work-up and management decisions.

This case report is written following the SCARE criteria [12].

## 2 Case presentations

Our first case is a 35-year-old female patient who presented with a sudden and persistent central abdominal pain of 24 h duration without relieving and aggravating factor. Associated with this she had loss of appetite, nausea and vomiting of ingested matter of similar duration. She gave history of abdominal swelling in the last 6 months. The patient didn't have any similar family history, no history of trauma to the abdomen.

Physical findings
On general appearance, the patient looked acutely sick
Vital signs: BP-90/50 PR- 120 and feeble
Abdominal examination: Diffuse tenderness all over the abdomen, and hyper tympanic to percussion
DRE: Empty rectum
On investigation

Full blood count mild leukocytosis with neutrophil predominance, and liver and renal function tests were within the normal range.

**Fig. 1:**
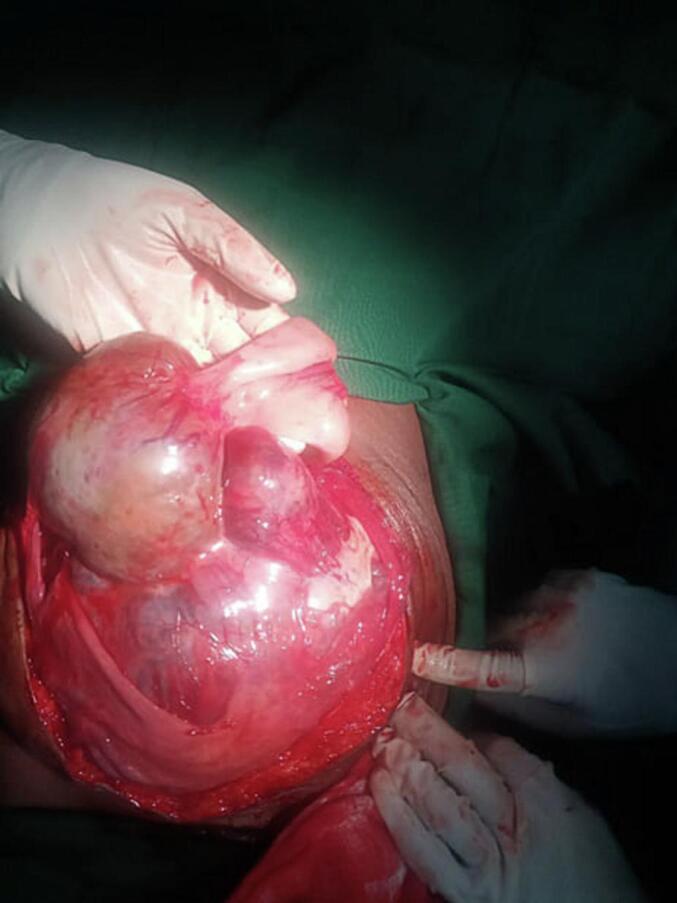
A huge abdominopelvic cystic mass adherent to the jejunum.

**Fig. 2:**
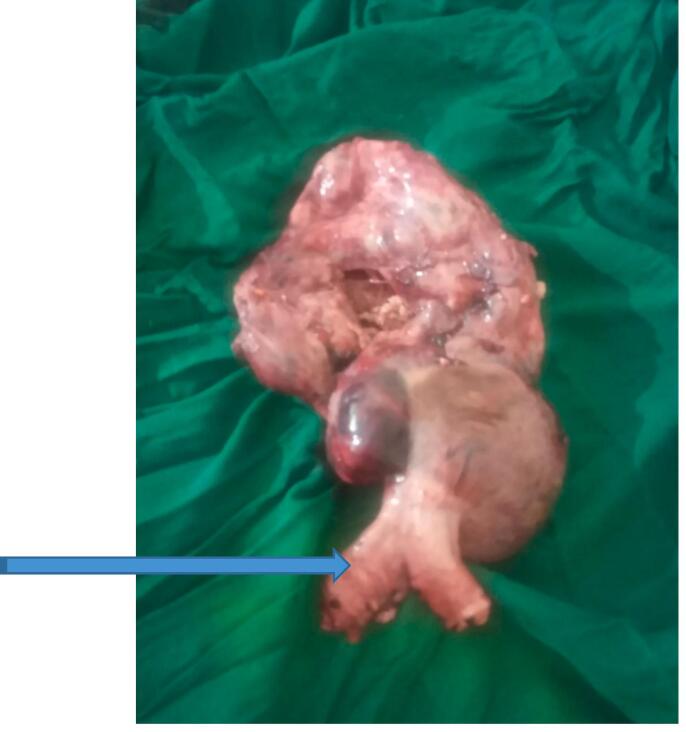
Resected mesenteric GIST (blue arrow indicating a portion of the jejunum that was resected in block). (For interpretation of the references to colour in this figure legend, the reader is referred to the web version of this article.)

Gangrenous small bowel obstruction was entertained. She was resuscitated with N/S and taken to the operation theatre after she produced adequate urine. Emergency laparotomy was done and the intraoperative findings were a huge abdominopelvic cystic mass adherent to the jejunum (Fig. 1) and causing mechanical obstruction. There was intracystic hemorrhage. The mass had loose adhesions with the ovaries and uterus with its broad base in the jejunal mesentry. The mass was excised with a portion of the jejunum and end to end jejuno-jejunal anastomosis was done (Fig. 2).

The specimen was sent for histopathologic examination.

**Fig. 3:**
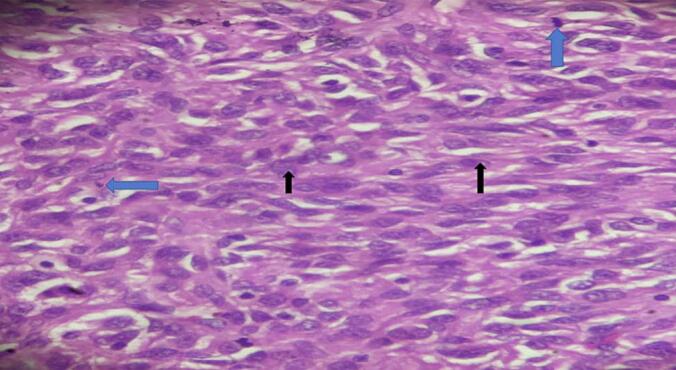
Solid sheets of epithelioid cells(black arrow) a long with frequent atypical mitotic(blue arrow). (For interpretation of the references to colour in this figure legend, the reader is referred to the web version of this article.)

The biopsy gross examination reviled serosally attached 15x14x4cm solid and cystic jejunal mass and the lesion's microscopic examination revealed solid sheets of epithelioid cells(black arrow) along with frequent atypical mitotic(blue arrow) and resection margins were free with the diagnosis of epithelioid high risk extra-GI mesenteric GIST (Fig. 3).

IHC was done for confirmation and CD117 become positive.

The patient had a smooth post-op course and discharged on her 4th post-op day. She came back for follow-up on her 2nd month of post-op. she was doing fine and linked to the oncology center to start imatinib.

The Second case was a 60-year-old male patient who presented with abdominal pain and vomiting of 5 days duration. The pain was more localized to the right hemiabdomen and the vomiting had bilious content. Associated with these he had failure to pass feces but can pass flatus. For the same complaint he visited our hospital a few weeks prior and was managed conservatively with the impression of partial small bowel obstruction and discharged.

#### 2.1.1 Physical examination

Acutely sick looking, he was tachycardic and febrile.

Abdomen- slightly distended abdomen, ill defined palpable mass at the right lower quadrant which was tender, hypertympanic to percussion, DRE-empty rectum, no mass, or blood on the examining finger.

**Fig. 4:**
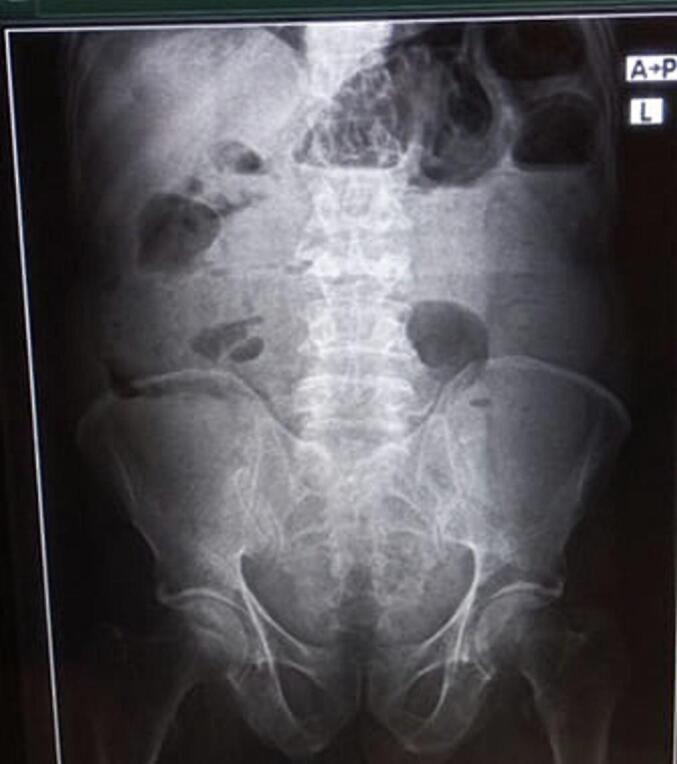
Plain abdominal X-ray of the patient suggesting small bowel obstruction.

**Fig. 5:**
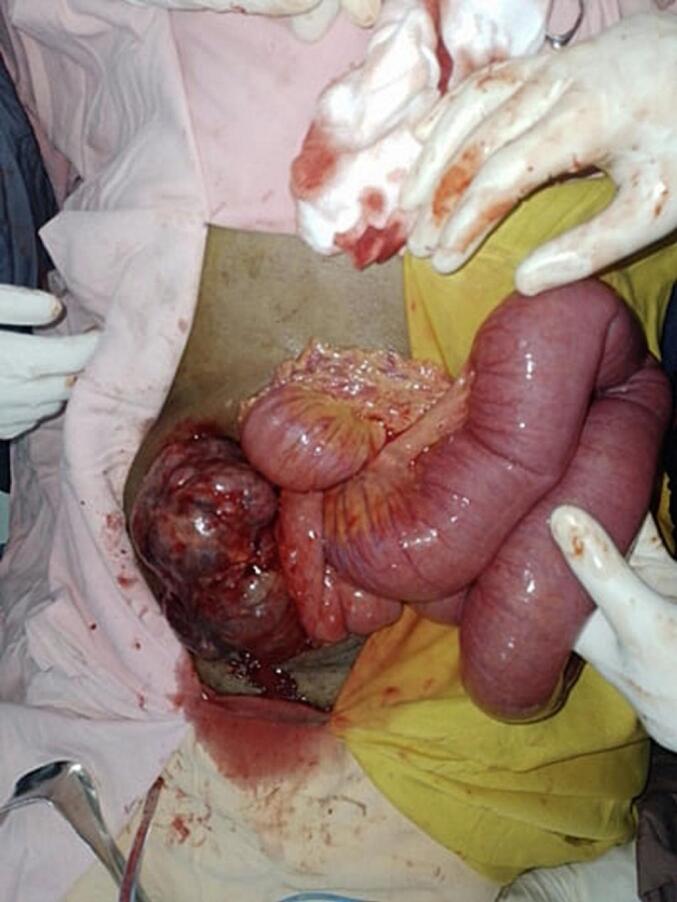
Distal jejunal GIST with distended proximal small bowel.

**Fig. 6:**
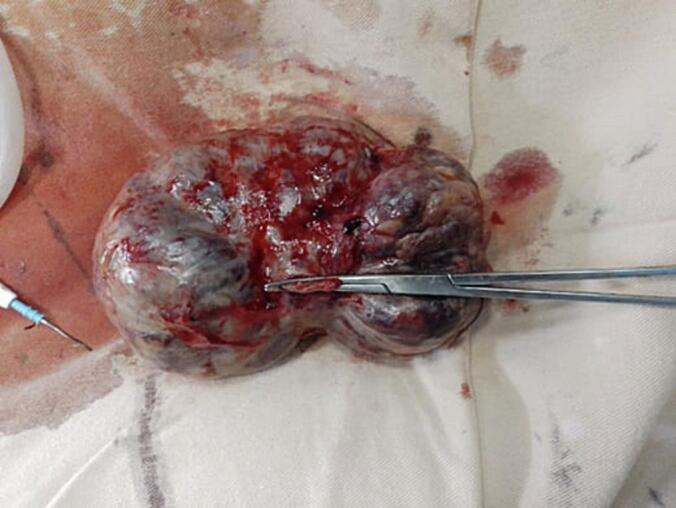
Resected specimen.

With the impression of small bowel obstruction secondary to small bowel mass, plain abdominal x-ray and abdominopelvic CT (Fig. 4) with contrast was done which suggested a distal jejunal exophytic mass causing a mechanical small bowel obstruction. The patient was resuscitated with normal saline, NG tube inserted, and catheterized. Once the patient produced adequate urine, he was taken to the operation theatre for exploratory laparotomy. Abdomen entered through a midline vertical incision. Intraoperatively, there was a huge jejunal mass in the right lower quadrant (black arrow) and the small bowel was significantly distended proximal to the mass. So, the mass was excised in block with adequate proximal and distal margins and end-to-end jejunal-jejunal anastomosis was done with double layer vicryl-3-0 (Fig. 5, Fig. 6).

**Fig. 7:**
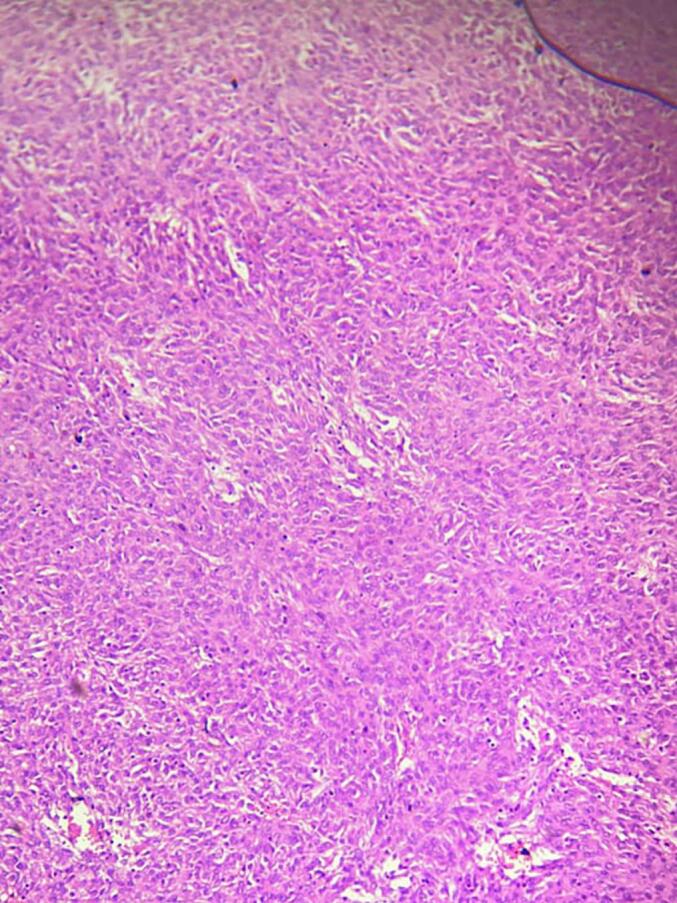
Solid sheets of epithelioid cells with prominent nucleoli a long with frequent atypical mitotic.

He stayed in the ward for two weeks and discharged home. He came to the follow-up clinic after two weeks with the histopathology result which showed a high grade epithelioid GIST(Fig. 7) for which we linked him to the oncology center.

The third case was a 72-year-old male patient who presented with epigastric pain of 1-year duration which worsened 3 month prior to his presentation. Associated with this he had intermittent vomiting of ingested matter 2× /week for the last 3 months and intermittent rectal bleeding(dark) for the last 3 months. He was being admitted and treated for dyspepsia at a local clinic several times. Before presenting to our hospital, he received 4 units of cross-matched blood in a nearby hospital.

On physical examination he was acutely sick looking in pain, he was tachycardic and hypotensive.

On abdominal examination- epigastric tenderness and mass was noted on the epigastric area.

He was investigated with upper GI endoscopy and conclude gastric volvulus or gastric intussusception.

**Fig. 8:**
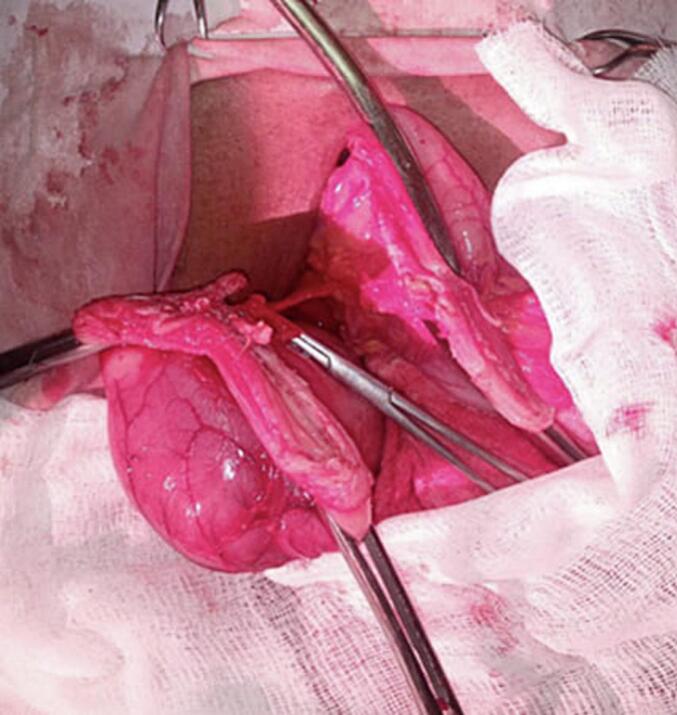
Divided stomach-partial distal gastrectomy.

**Fig. 9:**
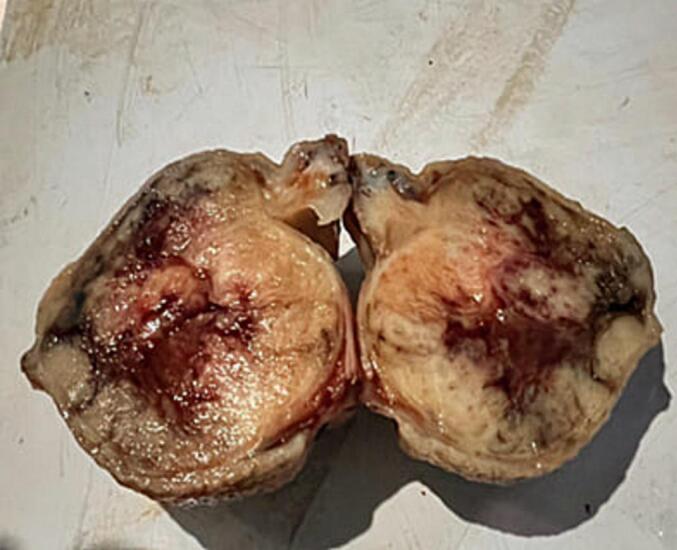
The resected gastric GIST.

**Fig. 10:**
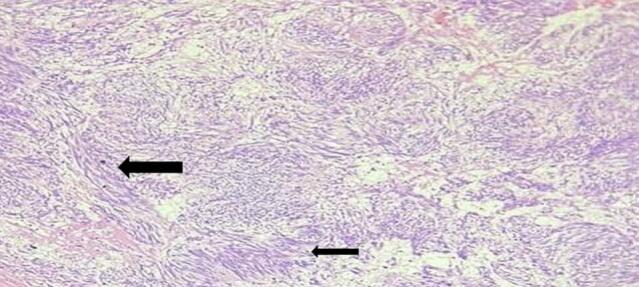
The patient was discharged on the 8th POD with smooth post op condition.

Abdominal CT was done and it showed enhancing gastric mass lesion (likely submucosal with endophytic growth) with invagination of gastric wall along the lesion and conclude gastro-gastric intussusception. With these impressions exploratory laparotomy was done after transfusing him with another 3 units of cross-matched blood. Intraoperatively there was pedunculated mass arising from the distal body and causing gastro-gastric intussusception. Distal partial gastrectomy was done. (Fig. 8, Fig. 9). The specimen was subjected to pathology and showed interlacing fascicles of spindle cells (black arrow) with conclusion of Low risk spindle GIST(Fig. 10).

The fourth case was a 55 yrs. old male patient who presented with vomiting of ingested matter of 3 months duration. The vomiting was intermittent and non-bilious. Associated with it he had an epigastric burning sensation and unquantified weight loss. He had no bleeding per mouth or anus. He had no family history of gastric cancer nor history of long standing peptic ulcer disease.

On physical examination he looked acutely sick looking with stable vital sign.

On HEENT- he had pale conjuctiva

On abdominal examination he had a huge intrabdominal mass localized to the epigastrium and right upper quadrant up to the umbilicus. There was no sign of fluid collection. DRE- no abnormality detected

#### 2.1.2 Investigations

Full blood count showed moderate anemia, and liver and renal function tests were within the normal range, hepatitis viral markers were negative.

**Fig. 11:**
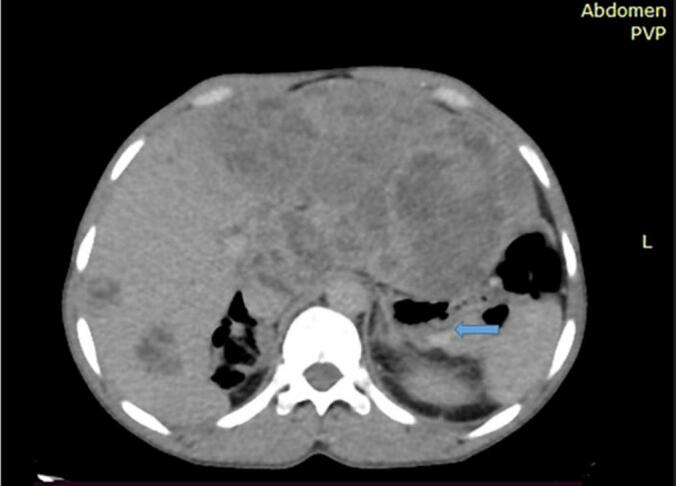
Ultrasound guided FNAC of the gastric mass and from the liver showed epitheloid GIST with secondary liver deposits.

Abdominal CT -multiple different sized mixed solid-cystic lesions on bilateral liver lobes and there is 6*5.4*5 cm measuring homogenously enhancing soft tissue lesion seen at the posterior wall of the body of the stomach, at the antral area having both exophytic and endophytic components (blue arrow) with conclusions of multiple necrotic liver lesions likely metastasis & exophytic gastric wall lesion likely GIST(Fig. 11).

The patient was admitted for possible exploratory laparotomy. Given he has symptoms of gastric outlet obstruction, our plan was to relieve this symptom either by doing a less radical resection or just to do a bypass. We gave him two units of X-matched blood before taking him to the operation theatre. We entered the abdomen through an upper midline incision; the liver was hugely enlarged and occupied the entire upper abdominal space. Upon exploring there was a mass arising from the posterior gastric wall very close to the duodenum. There was loose adhesion between the mass and surrounding visceral peritoneum. There was no ascites and no lymphadenopathies. We did wedge resection of the mass with adequate gross tumor free edge proximally but distally it was minimal. We took a liver biopsy. The patient had a good post-op course and discharge home on the 3rd post operative day. He came to our follow-up clinic after 3 weeks and during the visit his vomiting has subsided and he was relatively doing well. The biopsy result came back with the conclusion of a high risk epithelioid GIST with liver secondary. There was tumor involvement at the distal end. Then he was linked to the oncology center.

## 3 Discussion

GISTs are the most common mesenchymal tumors of the GI tract but make up only 0.1–3 % of all GI malignancies [10,13]. GISTs are hypervascular mesenchymal tumors which arise from interstitial cells of cajal, the cells which play a crucial role in regulating GI motility [5,6,14]. GISTs can arise anywhere along the GI tract [4,15].In our study, out of the four cases, two were gastric GISTs and one was jejunal. Small bowel GISTs are rare and jejunal GISTs are extremely rare (0.1–3 %) [4,15]. Jejunal GISTs accounts for 40 % of small bowels GISTs [4,5]. Unlike gastric GISTs, small bowel GISTs are difficult to diagnose, tend to rupture frequently, be more aggressive with poor prognosis, and require emergency surgery. They also tend to respond less to imatinib and tend to recur [6,13,15,16]. GISTs arising beyond the GI tract are extremely rare [12,17].These are termed as extra-intestinal GIST (*E*-GIST) [10,17]. E-GISTs commonly arise from the mesentry, omentum, or abdominal wall. They have similar radiologic and histopathologic features and are treated in the same manner like GISTs [10,17]. However, the overall survival of E-GISTs is much lower than GISTs [10,17]. Our first case was diagnosed to have an E-GIST which appeared to arise from jejunal mesentery.

GISTs commonly occur in men above the age of 50 years [1,7,13]. In agreement to this, three of our cases were male above the late fifties. Even though a vague abdominal pain is the most common symptom, the clinical presentation of GIST depends on tumor size and location [4]. All of our cases had a long-standing abdominal pain with repeated physician visits and unsettled diagnoses. GI bleeding is one of the characteristic features of GIST and nearly 64 % of small bowel GISTs present with bleeding. These bleedings are often occult [4,5,8,15]. In our study, all of the cases had varying degrees of anemia but the primary presenting symptoms of the case with jejunal GIST were symptoms of intestinal obstruction. In the literature, intraluminal growth of the GIST is incriminated as the primary mechanism of obstruction [14] but in two of our cases the obstruction was due to a mechanical compression of the bowels. An intussusception of a gastric GIST is a very rare phenomenon [5] but our third case presented with a gastric GIST with gastro-gastric intussusception. GISTs commonly metastasize to the liver (50–60 %) and peritoneum (20–43 %) and this is associated with a poor patient survival, 5 year survival of less than 25 % [2,4,8]. Our fourth case had a high-grade gastric GIST that metastasized to the liver.

Contrast enhanced abdominopelvic CT scan plays a major role in diagnosis, staging, and follow-up of GISTs, particularly in case of small bowel GIST. This is because of a big challenge in localizing the tumor using standard endoscopy [1,14,15]. MRI and PET scan are the other imaging modalities which can be used to detect small GISTs and possibly consider neoadjuvant treatment [3,10]. GISTs are distinguished from other tumors using immunohistochemical test positivity for CD 117, CD 34, and several others like S-100 and DOG-1. About 90–95 % of GISTs show CD117 positivity [1,10].In our study all of the cases have shown positivity for CD 117.

The predominant histological pattern of GIST is spindle-cell type (70 %) which appears as fusiform cells in intersecting whorls. The other less common patterns are epithelioid types (20 %), appearing as rounded cells in a nested pattern, and rarely mixed pattern [7,8]. Studies have shown that mixed variants have a poor prognosis [16].Three of our cases had an epithelioid morphologic pattern and the spindle type was noted in only one patient. In general, GISTs have benign behavior but size of the tumor and mitotic count are important indicators of aggressiveness and risk of metastasis of a GIST [6,8]. Depending on these two factors GISTs are classified as very low, low, intermediate, and high risk. Those labeled as high risk have a high risk of recurrence and require adjuvant therapy [6,8,10]. In our study, three of the cases had a high-risk GIST.

To have a good patient outcome, early diagnosis and treatment of GIST is very important [6]. Radical surgical resection is the cornerstone of treatment of GIST followed by risk stratification [4]. Adjuvant tyrosine kinase inhibitors like imatinib mesylate therapy is indicated for high risk GISTs [4,6]. Those with low risk GISTs can be followed for any recurrence [14]. In our study all of them underwent resection and three of the cases were linked to oncology center for possible initiation of Imatinib as they had a high-risk GIST.

## 4 Conclusion

Even though GIST constitutes the smallest portion of GI neoplasms, physicians need to have high index of suspicion for it in patients presenting with vague abdominal pain and consider abdominopelvic CT as part of patients' work up. IHC examination is crucial in confirming the diagnosis of GIST and clinicians should subject resected specimens for this test. Adjuvant Imatinib should be considered in all patients with high risk GIST.

## Author contribution

Alazar Berhe Aregawi MD- Study concept and design, literature review of the patient, editing and critical review of the paper, treating the case.

Bizunesh Dires Fenta, MD - Study concept and design, editing and critical review of the paper, diagnosing the case.

Mekashaw Altaseb Worku, MD - Involved in acquisition of data and managing the patient.

Teketel Tadesse Geremew, MD - literature review of the paper, writing and drafting the paper, editing and critical review of the paper.

## Informed consent

The report was done with patient's consent.

## Ethical approval

A case reports encountered incidentally and not involving experimental intervention do not require formal ethical approval.

## Guarantor

Teketel Tadesse Geremew, MD.

## Funding statement

“No funding was used in this study.”

## Declaration of competing interest

No conflicts of interest.

## Data Availability

The data used to support the findings of this case report are available from the corresponding author upon reasonable request.
